# Non-invasive transmission of sensorimotor information in humans using an EEG/focused ultrasound brain-to-brain interface

**DOI:** 10.1371/journal.pone.0178476

**Published:** 2017-06-09

**Authors:** Wonhye Lee, Suji Kim, Byeongnam Kim, Chungki Lee, Yong An Chung, Laehyun Kim, Seung-Schik Yoo

**Affiliations:** 1 Incheon St. Mary’s Hospital, The Catholic University of Korea, Incheon, Korea; 2 Department of Radiology, Brigham and Women’s Hospital, Harvard Medical School, Boston, MA, United States of America; 3 Center for Bionics, Korea Institute of Science and Technology, Seoul, Korea; Shanghai Jiao Tong University, CHINA

## Abstract

We present non-invasive means that detect unilateral hand motor brain activity from one individual and subsequently stimulate the somatosensory area of another individual, thus, enabling the remote hemispheric link between each brain hemisphere in humans. Healthy participants were paired as a sender and a receiver. A sender performed a motor imagery task of either right or left hand, and associated changes in the electroencephalogram (EEG) *mu* rhythm (8–10 Hz) originating from either hemisphere were programmed to move a computer cursor to a target that appeared in either left or right of the computer screen. When the cursor reaches its target, the outcome was transmitted to another computer over the internet, and actuated the focused ultrasound (FUS) devices that selectively and non-invasively stimulated either the right or left hand somatosensory area of the receiver. Small FUS transducers effectively allowed for the independent administration of stimulatory ultrasonic waves to somatosensory areas. The stimulation elicited unilateral tactile sensation of the hand from the receiver, thus establishing the hemispheric brain-to-brain interface (BBI). Although there was a degree of variability in task accuracy, six pairs of volunteers performed the BBI task in high accuracy, transferring approximately eight commands per minute. Linkage between the hemispheric brain activities among individuals suggests the possibility for expansion of the information bandwidth in the context of BBI.

## Introduction

Brain-to-Computer-Interface (BCI) techniques detect neural signals associated with brain function and translate them to computer/machine control commands. Conversion of the commands to stimulate specific brain regions of other individuals allows for the formation of the Brain-to-Brain-Interface (BBI) that directly links the specific brain activities among individuals/biological entities. In this context, brain stimulation modalities controlled by a computer are considered as the Computer-to-Brain Interface (CBI). Capitalizing on internet connectivity of computers as an intermediary, BBI has the potential to transmit neural information over long distances with a wide information bandwidth.

Direct cortical recording/stimulation achieved by implanted electrodes has been used to obtain neural signals as well as to deliver stimulation to the designated brain regions among rodents [1–5] and non-human primates [6–10]. These implantable devices can be used for achieving BBI; however, the risks associated with the surgical procedure for the electrode implantation limit its applicability to humans. Therefore, to be compatible with experimentation in healthy humans, non-invasive procedures need to be considered for both BCI and CBI modalities.

Various BCI systems have been developed and become widely available based on non-invasive neural recording/neuroimaging techniques such as surface electroencephalography (EEG) [11], magnetoencephalography (MEG) [12], and functional magnetic resonance imaging (fMRI) [13]. For CBI portion of the implementation of BBI, non-invasive brain stimulation techniques such as transcranial magnetic stimulation (TMS) [14, 15] or transcranial focused ultrasound (FUS) [16–18] are available for translating the digital information from a computer to stimulate a certain region of the brain.

Since the initial theoretical conceptualization [19–21], we have witnessed the actual implementation of the non-invasive BBI techniques in recent years. For example, we have shown that human intention, as detected by the EEG steady-state visual evoked potentials (SSVEP), can be translated to stimulate the rodent brain of the tail motor area using transcranial FUS [22]. Li and Zhang have reported interfacing human brain function, driven by the SSVEP, to control insect behavior by electrically stimulating their antennae [23]. Grau and colleagues used the binary coding of motor imagery-mediated EEG potentials from a human subject to remotely actuate a TMS device that stimulated the visual areas of another human individual, thus, granting the transmission of motor imagery intention between the subjects [14]. In the same year, Rao and colleagues used motor imagery EEG signals from an individual playing a simple computer game, and stimulated the motor cortex of another subject (to push a ‘fire’ button) who was simultaneously playing the same game using TMS [15]. The same group further applied a similar technique, combined with a traditional motor task (manipulation of a computer mouse), to allow mutual interactions between two individuals in solving a Twenty Questions game [24].

TMS is an established non-invasive brain stimulation modality, and is the preferred choice as a non-invasive CBI. Although the affected area by the TMS stimulation is on the order of 0.5–1 cm [25], better spatial selectivity is warranted. Also, TMS is often accompanied by undesired tactile sensations from the scalp (through activation of skin tactile receptors) and loud clicking sounds (due to the pulsed application of strong magnetic field) [26–28], which make a confounder-free study design difficult to attain. In addition, one must consider the presence of mutual interference/distortion/interaction of the magnetic field, especially when multiple TMS coils are to be placed in close proximity and actuated simultaneously [29, 30].

Transcranial FUS technique, on the other hand, uses a highly-focused formation of acoustic pressure waves to stimulate the neural tissue including the deep brain structures, and has small neuromodulatory areas (on the order of millimeters in diameter). The acoustic energy can selectively reach deeper target areas than TMS. Although the exact mechanism behind the stimulation is not clearly elucidated, the effects of FUS have been associated with mechanical coupling between the acoustic pressure waves to various neural components, which ramifies into changes in membrane capacitance, mechanoreceptor-mediated action potentials, neuron-glia interactions, and/or neurotransmission [16, 17, 31–33]. This is contrasted to the mechanism of TMS (also not clearly understood), whereby the application of a strong magnetic field induces electrical currents in underlying neural tissue.

FUS has recently been tested to elicit tactile sensations from the hand area by stimulating the primary somatosensory area (SI) of the human brain [16]. It has also been used in modulating visuomotor function of non-human primates [34] as well as in changing sensations associated with passive tactile stimulation [18]. Due to a small footprint of the FUS transducer and the non-electromagnetic operation in ultrasound sonication, mutual electromagnetic interference does not occur between multiple transducers. Recently, we have shown that both SI and secondary somatosensory area (SII) on the same side of the brain hemisphere can be successfully stimulated using two separate FUS transducers [35]. Therefore, selective and separate stimulation of a brain region in the left and right hemisphere can be readily performed, all of which indicate the utility of FUS as a new mode of non-invasive CBI.

In the present study, we detected the neural function associated with two directional (right or left) motor imagery choices using EEG-based BCI from human participants (as a sender), and relayed the information to selectively stimulate either the right or the left SI of the hand from another individual (as an information receiver) by independent operation of a two set of FUS transducers. Subsequently, the timing of tactile sensation (elicited in either the left or right hand) was reported through finger-tapping of the corresponding hand. The region of FUS stimulation was image-guided based on individual-specific functional neuroanatomy. Two individuals were separated by a large distance (~30 km) and information packets were transmitted over the internet to achieve BBI implementation.

## Materials and methods

### Overview of the study design

This research was conducted in accordance with the approved guidelines set forth by the Institutional Review Boards (IRBs) of two following participating institutions: the Korea Institute of Science and Technology (KIST 2014–008; where the BCI procedure was conducted) and the Incheon St. Mary’s Hospital, the Catholic University of Korea (where the CBI procedure was conducted). These institutions exchanged the contents of the respective IRB review and jointly approved the research.

**Fig 1:**
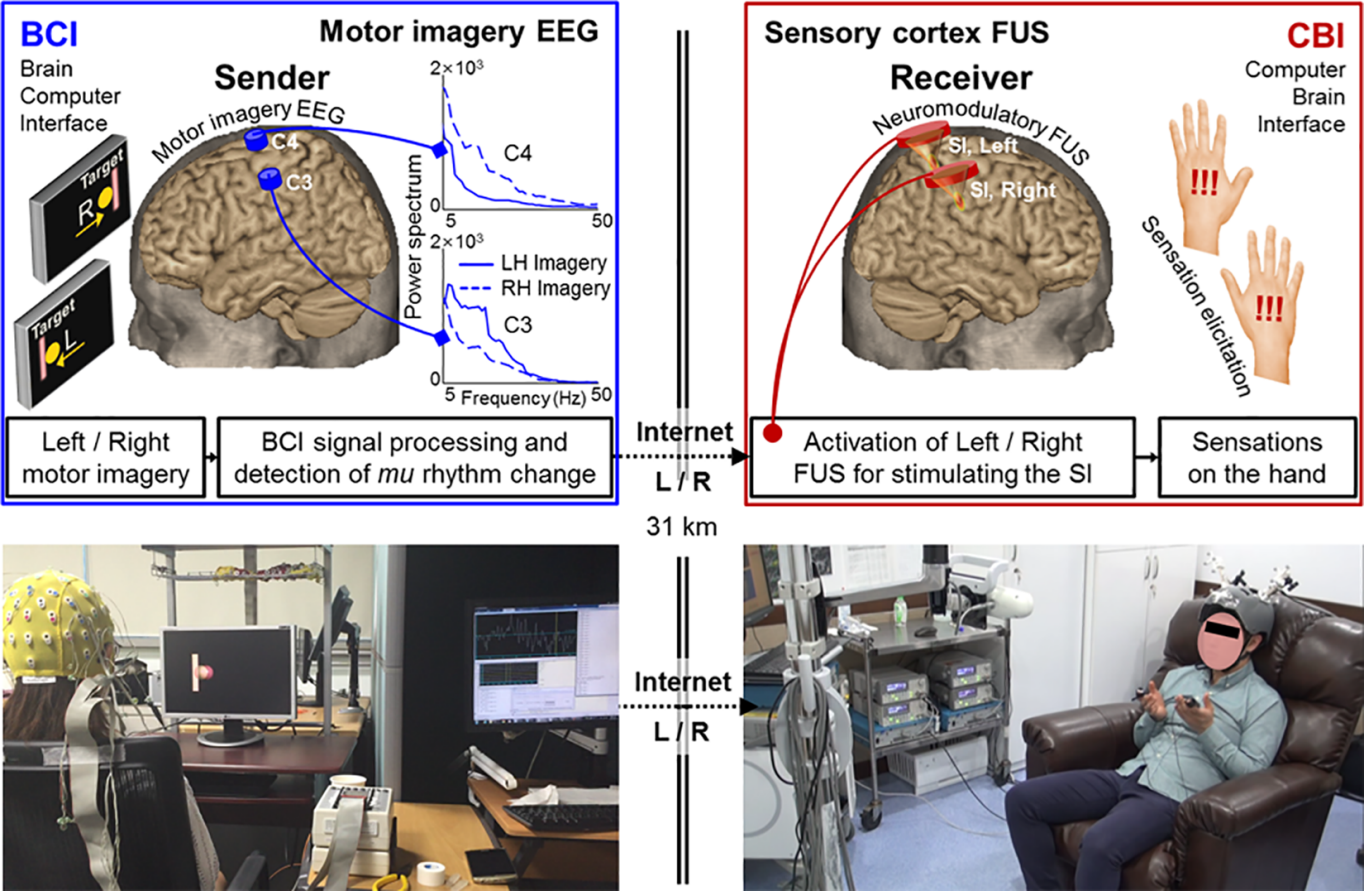
Schematics and exemplar view of the brain-to-brain interface (BBI) system. On the left panels, the EEG-based BCI procedure is shown, including the placement of EEG electrodes over the motor cortices. Upon the presentation of the left/right target on a computer display, the sender’s motor imagery of the left/right hand modulates the EEG signal through the reduction of the *mu* rhythm in each hemisphere (*i*.*e*., illustration given in the C3 and C4 EEG location), and moves the computer cursor (appeared as a ball) through BCI signal processing. When the cursor reaches its target, the computer generates a trigger signal that is transmitted to the receiving location (~30 km away) through the internet (*via* TCP/IP protocol), whereby the FUS-based CBI (illustrated on the right panels) stimulates either the left or right SI. The right panel illustrates implementation of two FUS transducers that independently stimulate left/right SI of the receiving individuals. The stimulation of the SI elicits tactile sensation of the contralateral hand area, and the receiver was instructed to signal the hand where she/he felt the elicited tactile sensations.

The overall study design for the implementation of the BBI is briefly described (in **Fig 1**). An individual (as an information sender) participating at one institution (KIST, Seoul, Korea) underwent a motor imagery-based BCI procedure to move a computer cursor in either the left or right direction. EEG was used to detect the modulation of *mu* (μ) rhythms originating from corresponding hand motor brain areas of each hemisphere, and the degree of modulation was translated to move a ball cursor to the target. Depending on the outcome, one of the following computer commands—‘Left’, ‘Right’, ‘Miss’, or ‘Abort’ were generated (where ‘Miss’ indicates the false command generation and ‘Abort’ indicates the failure to generate any commands in the designated time window) and subsequently relayed to another computer located in the other institution (Incheon St. Mary’s Hospital, ~30 km away), where the receiver underwent a FUS-based CBI procedure. Transmission control protocol (TCP)/internet protocol (IP) was used for transmission. The receiver computer activated the FUS devices that can selectively stimulate the left or right hand SI area, and CBI participants were instructed to press the thumb against their index finger on the same side (*i*.*e*., left or right) where they felt tactile sensations on their hand/arm area. The motion was recorded by touch sensors (pulse transducer MTL1010/D; ADInstruments, CO) attached to their index fingers. Three separate sessions of BBI were conducted while each consisted of 20 task trials.

### EEG-based BCI implementation

#### Participants

For the EEG-based BCI, 29 right-handed healthy volunteers (age = 22.3 ± 4.5; mean ± s.d., range 19–32, 11 females) were initially recruited. All the participants were free from any neurological illness based on a self-reported survey. Handedness was determined by a personal interview and review of handedness criteria based on the Edinburgh handedness inventory [36]. The participants gave their written consent prior to the commencement of the experiment.

#### EEG BCI data acquisition and processing

EEG signals were acquired using a standard 64-channel active electrode EEG system (the ActiveTwo, BioSemi Systems; Amsterdam, The Netherlands) with a sampling rate of 2048 Hz. Among the 64 channels, eight channels (F3, F4, C3, C4, Cz, T7, T8, and Pz) were used for the EEG recording and the signals acquired from the C3 and C4 were selected for the analysis of motor imagery EEG. The two channels (C3 and C4) reflect the brain activity from the left and right motor cortices, respectively, which represents the right and left hand motor imagery [37]. The common mode sense (CMS) channel located posterior to the Cz in the parietal cortex was used as a reference channel. A BCI2000 platform was used for processing the EEG data and for generating the BCI commands [38]. The software configurations were adjusted to be compatible with the EEG acquisition system, and power spectra were calculated using autoregression (using ‘ARsignalProcessing module’ of BCI 2000) [38].

#### Test sessions for setting the BCI parameters and subject selection for the BBI

The BCI task performance was examined among the recruited participants, prior to the BBI session, because high degrees of variations exist in subject-dependent success rate for performing BCI tasks [39]. The participants were seated on a comfortable chair and instructed to watch the computer screen (having a size of 22 inch with a viewing angle of ~15 °) (**Fig 1**). The distance between the center of the chair and the screen was approximately 45 cm.

**Fig 2:**
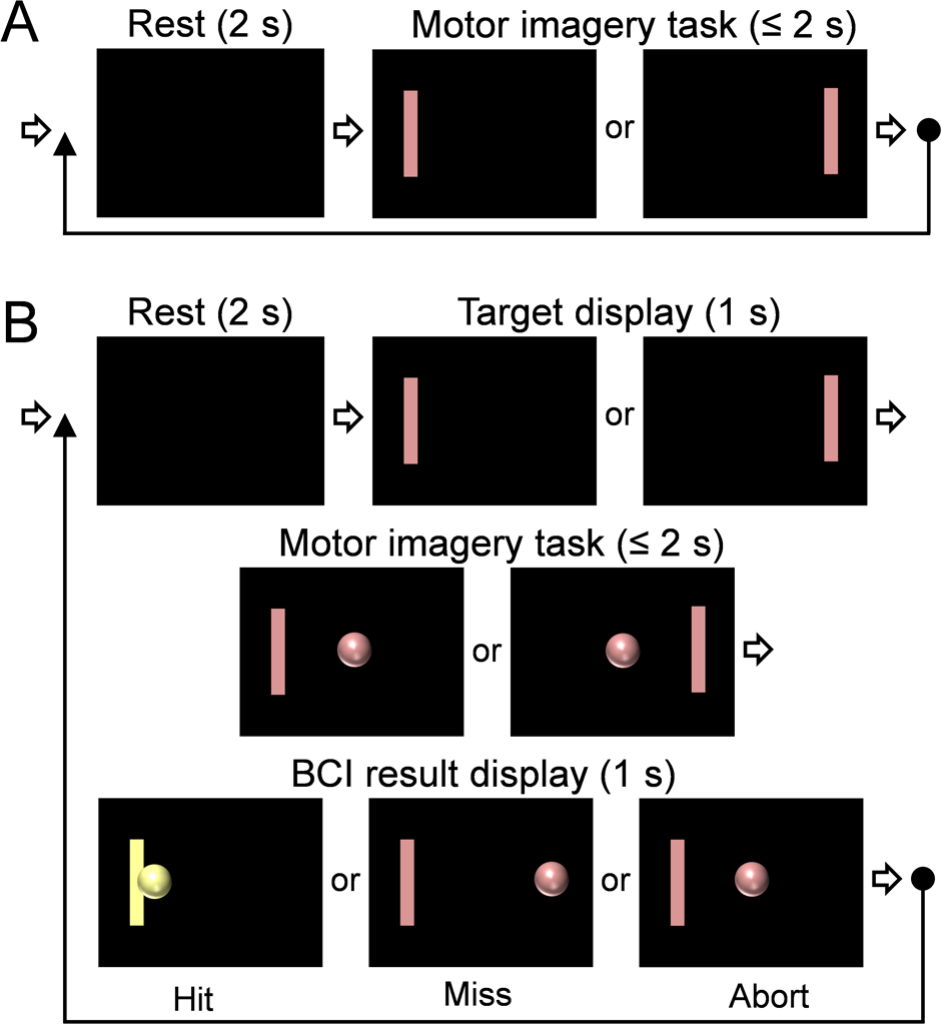
Flow charts of the visual displays used for the brain-computer interface (BCI) task trials. (**A**) The timing diagram of BCI test sessions used for setting the BCI parameters. (**B**) The timing diagram used for BCI experiment session for decoding the motor intention during the BBI implementation.

A set of three BCI test sessions, each consisting of 20 task trials (using the ‘Stimulation Presentation module’ of the BCI 2000), were conducted to acquire enough EEG data to generate subject-specific parameters. In each trial, a target bar (**Fig 2A**, in pink) was presented on either the right or left side of the computer screen for 2 s. Each participant was asked to perform a motor imagery task (clenching of either the left or right hand) depending on the side of the target on the screen until the target bar disappears (**Fig 2A**). The motor imagery task was repeated 20 times, with a resting period of 2 s in-between. The side of the targets were randomized and balanced (having 50% chance of being left or right). Also, the participants were discouraged from making eye blinks and tongue movements during the motor imagery period to avoid motions that decrease the signal-to-noise ratio (SNR) of the EEG signal [40].

The EEG signals from the C3 and C4 were used to generate subject-specific parameters for decoding the motor intention. A power spectrum was generated from the EEG data using an autoregressive model [38] whereby a bandpass filter of 0–30 Hz, having a bandwidth of 1 Hz, was used while the order of autoregression was set to 16. The frequencies within the alpha band (8–14 Hz) that showed the most significant spectral power differences between the two channels were identified during the training session, and the amplitude gain for each spectral frequency was adjusted to balance the amplitude between the two. Four frequency bands, 8, 10, 12, and 14 Hz, were used for motor imagery classification to detect the *mu* rhythm change associated with the imagery task. We used the automatic gain-setting feature in the BCI2000, using the normalizer algorithm that estimates offset and gain values adaptively to make its output signal achieve zero mean and unit variance. For the spectral estimation parameters, the bin width of 1 Hz and Hamming window (bandwidth of 0.5 s) were used. The Hamming window setting was chosen to update the classification results frequently although a longer (*i*.*e*., 1 s) Hamming window setting could offer a higher SNR. Finally, the identified frequencies and the gain were saved as a parameter setting for subsequent BCI sessions.

#### BCI experiment session for decoding the motor intention

The subject-specific parameter generated during the test BCI sessions was used as a linear classifier. If needed, the gain of each frequency and channel was adjusted further. The protocol for the BBI experiment session consisted of four consecutive periods: rest (2 s), target display (1 s), motor imagery task (within 2 s), and BCI result display (1 s) (**Fig 2B;** using the ‘CursorTask module’ of BCI 2000). Three BCI experiment sessions were performed, while each session consisted of 20 task trials. A task trial began with the rest period where a blank screen was shown. Then, the target bar appeared at the left or right side on the screen for 1 s in a randomized order (target display period). Subsequently, the subject was asked to evoke imagery, which entailed clenching the hand that corresponds to the side of the left/right target without executing an actual motion (randomized and balanced; *i*.*e*., 10 left and 10 right targets). During this motor imagery task period, the cursor (represented by a ball cursor) was presented at the center of the screen in the beginning, and the participant was asked to perform the motor imagery task until the ball cursor hits the target bar. The position of the ball cursor was controlled by the output of a linear classifier depending on the relative power difference between the EEG frequency bands specifically calibrated for each participant.

The movement of the ball cursor gave the visual feedback to the subject about how the BCI task was executed. When the ball cursor reached the target within 2 s, the result (Hit; color-coded as yellow) was briefly displayed for 1 s (period of result display). Then, the rest period of the subsequent trial initiated (an example shown in **Fig 2B**). In case that the cursor reached the opposite end (from the intended side of the target), the result was categorized as ‘Miss.’ If the ball cursor did not reach the target within 2 s and floated on the screen between the targets, then it was categorized as ‘Abort’. There was no color change displayed for 'Miss' and 'Abort.' Only seven individuals showed the task accuracy greater than 70%, and six participated for the subsequent BBI experiment (age = 25.9 ± 7.8; mean ± s.d., range 19–32, two females, named as ‘BC1’ through ‘BC6’ herein).

### Interface between BCI and CBI

The result of each trial (‘Hit’, ‘Miss’, or ‘Abort’) from BCI experiment session was sent to a custom-built MATLAB program (Mathworks, Framingham, MA) installed on the computer in the BCI segment, and then was sent to another computer (in the CBI segment) through TCP/IP communication as a text packet (a string variable having a data size of 192 bit). Each communication packet contained the trial number, the task timing, the BCI task results, and the hemispheric side of the CBI stimulation. For example, a string data of 'T010 11:14:58.38 H R' means that the packet for the 10th task trial (*i*.*e*.,’T010’ portion) was sent to the CBI segment at 11:14:58.38 (time stamp). ‘H’ and ‘R’ correspond to the result of ‘Hit’ and the hemispheric side of the CBI stimulation (as right), respectively. In case the left target was missed during the BCI segment, CBI stimulation was given to the right hemisphere (an example of a generated string would be ‘T02 18:30:20.48 M R’). The time stamp was generated with respect to the standard local time provided by a network time synchronization software (UTCk3.1; Korea Research Institute of Standards and Science), whereby the timing of the two computers at the BCI and CBI segments were synchronized before the BBI sessions. Upon receiving the text packet, the MATLAB scripts installed on the CBI computer generated a trigger signal for the programmed operation of the FUS device that selectively stimulates either the right or left SI area independently (see below ‘Sonication setup and Acoustic parameters’ section).

### FUS-mediated CBI implementation

#### Participants

Six healthy volunteers (age = 28.2 ± 9.5; mean ± s.d., range 23–45, one female, named as ‘CB1’ through ‘CB6’ herein), free from clinical history of central/peripheral nerve diseases, participated in the FUS-based CBI segment of the experiment, and gave written consent prior to the study participation. The demographics were not matched between the subjects participating in either BCI or CBI segments of the experiment.

#### Multi-modal imaging acquisition and processing for sonication planning

Doughnut-shaped adhesive fiducial markers (PinPoint; Beekley Corp., Bristol, CT), which are visible in both MRI and CT, were attached on four different locations, over the skin of the forehead and back of the ears, as described previously [16]. The locations of the attached fiducial markers were later utilized to co-register the spatial coordinates of the subject-specific neuroimaging data with the actual head anatomy. Then, the participants underwent anatomical and fMRI sessions to map their left/right hand SI. A clinical 3 Tesla MR scanner (MAGNETOM Skyra, Siemens) was used to acquire the anatomical and functional MRI data from the participants’ brains using a 4-channel head coil. First, T1-weighted images (3D GRAPPA sequence, acceleration factor = 2, TR/TE = 1,900/2.46 ms, flip angle = 9°, slice thickness = 0.94 mm, field-of-view [FOV] = 24 × 24 cm^2^, image matrix = 256 × 256, voxel size = 0.94 × 0.94 × 0.94 mm^3^) from the entire telencephalic area of the head was obtained in the sagittal orientation for the brain anatomical information. Computed tomography (CT) data were acquired using a clinical CT scanner (Aquilion ONE, Toshiba, Japan) from the participant’s head (axial orientation, slice thickness = 0.5 mm, FOV = 24 × 24 cm^2^, image matrix = 512 × 512, voxel size = 0.47 × 0.47 × 0.50 mm^3^). This was done to examine the presence of intracranial calcification and to have the information of the skull structures for planning the incident angle of the transcranial sonication to the SI.

To identify the individual-specific eloquent functional area of the hand SI, fMRI was conducted using a gradient-echo echo-planar-imaging (EPI) sequence (TR/TE = 2,500/30 ms, flip angle = 90°, slice thickness = 4 mm, FOV = 24 × 24 cm^2^, image matrix = 96 × 96, voxel size = 2.5 × 2.5 × 4 mm^3^) with an oblique scanning orientation, having the image slices parallel to the line connecting the anterior commissure (AC) and the posterior commission (PC) of the brain. For the fMRI data acquisition, three blocks (25 s-long) of hand-clenching task (clenching one hand approximately twice per second) were interleaved by four blocks of resting period with equal duration. The task timing was provided by the visual cue delivered *via* an MRI compatible screen (E Sys fMRI, Invivo, Gainesville, FL). All the subjects underwent separate fMRI sessions for mapping the left and right hand SI, respectively. The acquired fMRI data were analyzed using the SPM8 software (Wellcome Department of Imaging Neuroscience, University College London, London, UK; www.fil.ion.ucl.ac.uk/spm), whereby a general linear model (GLM) was used to estimate the task-related neuronal activity after the motion correction. Following the GLM analysis, a voxel-wise statistic parametric map (in *t*-value), with respect to the canonical hemodynamic response function (HRF), was obtained using a threshold of *P* < 0.05 (family-wise error [FWE] corrected) for the visualization of the blood-oxygenation-level-dependent (BOLD) activations. The local maximum of the activated area posterior to the central sulcus within the postcentral gyrus was set as the sonication target for the FUS sessions. The multi-modal imaging data from the anatomical MRI, functional MRI, and CT were co-registered based on the normalized mutual information [41], and utilized in the image-guided FUS targeting for the CBI segment. The acquired MRI and CT data were also used for neuroradiological assessments to examine any neuroanatomical abnormalities or clinically-significant intracranial calcifications (none were found).

#### Sonication setup and acoustic parameters

**Fig 3:**
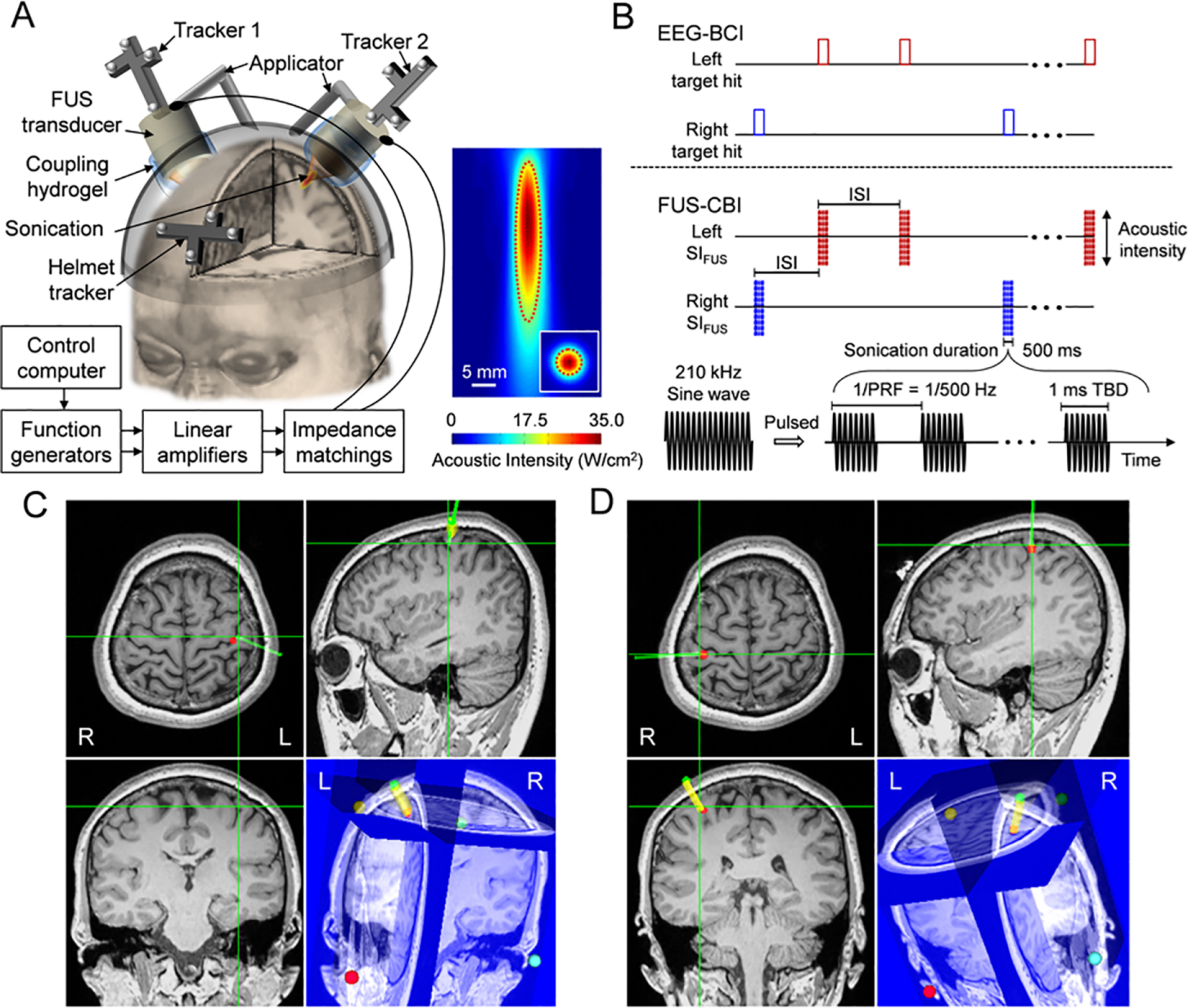
Schematics for the FUS-mediated CBI system in BBI communication. (**A**) Left panel: a rendering of the FUS setup for the CBI segment. The left and right SI were independently targeted by two single-element 210 kHz FUS transducers where the locations of FUS foci were tracked by using the optical trackers (‘tracker 1’ and ‘tracker 2’) in reference to the head (tracked *via* ‘helmet tracker’). Each tracker having four infrared-reflective markers was recognized by a motion capture camera. The gap between the transducer and the scalp was filled with a soft compressible hydrogel for the acoustic coupling. FUS transducers were actuated by separate sets of computer-controlled driving circuits. Right panel: The acoustic intensity mapping of the FUS transducer. The FWHM of the intensity profile is demarcated by red dotted lines. (**B**) Illustration of the FUS actuations triggered by the transmitted signals from the BCI segment with the following acoustic parameters: SD = 500 ms; ISI, inter-stimulation-interval, = 7 s; TBD = 1 ms; PRF = 500 Hz; I_sppa_ = 35.0 W/cm^2^. (**C** and **D**) Exemplar views of the FUS targeting to the bilateral SI as guided by individual-specific neuroimage data. The sonication target is indicated as the intersection of the green orthogonal crosshairs shown in the triplanar views (*i*.*e*., axial, sagittal, and coronal slices), while the thick yellow line (connecting green and red dots as intended entry to target points) and green line represent the orientation of the planned and guided sonication beam paths, respectively. In the lower right panel of 3D rendered view, the four colored dots show the locations of anatomical markers used for the spatial co-registration between the subject and the neuroimage data. ‘R’ and ‘L’ denote right and left, respectively.

Two air-backed, single-element 210 kHz FUS transducers (Ultran, PA; outer diameter of 30 mm and focal distance of 25 mm) were used to deliver the acoustic energy to the left/right hand SI of the brain (**Fig 3A**). Each transducer was connected with an articulated arm mounted on a customized helmet setup having open spaces over the bilateral hand SI. By using the articulated arms, the location and orientation of the FUS transducers were adjusted to align the foci to the left and right hand SI areas, as guided by neuroimage data (details described in ‘FUS navigation and guidance for the CBI segment’ below). The acoustic path from the transducer surface to the scalp was coupled by a compressible polyvinyl alcohol (PVA) hydrogel, having a preformed shape to be abutted on the curvature surface of the transducer [42]. The hair was carefully combed away from the entry point of the sonication path, and a generic ultrasound hydrogel (Aquasonic; Parker Laboratories, Fairfield, NJ, USA) was applied on all the interfaces among the transducer, PVA hydrogel, and the exposed scalp. For the image-guided FUS targeting, optical trackers having four infrared-reflective markers, which can be recognized and tracked by a motion capture camera (Vicra, Northern Digital, Ontario, Canada), were attached to the anterior part of the FUS helmet and the back of the FUS transducers (illustrated in **Fig 3A**) [16].

The spatial profile of the acoustic intensity (AI) around the FUS focus was characterized using the methods described elsewhere [43] and shown in **Fig 3A**. The diameter of the focus was estimated on the transversal plane (31 × 31 mm^2^ square area, 1 mm step) perpendicular to the sonication path at the focal distance (based on the time-of-flight information), and the length of the focus was measured on the longitudinal plane along the beam path (31 × 51 mm^2^ rectangular area, 1 mm step). The acoustic focus has a diameter of 6 mm and a length of 38 mm, as defined by the full-width at half-maximum (FWHM) of the acoustic intensity map. The incident intensity at the FUS focus had a spatial-peak pulse-average AI (I_sppa_) of 35 W/cm^2^, resulting in a spatial peak temporal-average AI (I_spta_) of 17.5 W/cm^2^.

For the actuation of each FUS transducer, the input signal was generated by using function generators (33220A; Agilent technologies, Inc., Santa Clara, CA) and amplified by a class-A linear power amplifier (Electronics and Innovations, Rochester, NY) with an impedance matching circuit. Two separate sets of the circuit setups were prepared to selectively actuate the FUS transducers (**Fig 3A**). During the later BBI sessions, the function generators were triggered to generate the signals by a local control computer (in the Incheon St. Mary’s Hospital) when the computer received the transmitted communication packet (having left/right information) from the BCI segment through TCP/IP communications (**Fig 3B**). For the FUS stimulation of the SI, we adapted the following sonication parameters that were used in our previous studies of the brain stimulation in human SI [16]: a tone-burst-duration (TBD) of 1 ms with a pulse repetition frequency (PRF) of 500 Hz (*i*.*e*., duty cycle of 50%), and a sonication duration (SD) of 500 ms (**Fig 3B**). Based on the 50% duty cycle and the rate of acoustic transmission (20–25%) to the intended targets of the SI [16], the energy level delivered to the targeted brain areas was estimated to be 3.5–4.4 W/cm^2^ I_spta_. In our previous study [35], using the same sonication settings of ultrasound transducers and parameters, the FUS was safely administered to the human SI as well as SII (across all ten participants). Also, the potential thermal increase at the sonicated region of the brain was negligible (estimated to be < 0.01 °C [35]), which is much lower than the previously reported thermal threshold for inducing tissue damages or temperature-mediated stimulatory effects in the brain [44, 45].

#### FUS navigation and guidance for the CBI segment

The subject in the CBI segment was seated on a recliner chair. Fiducial markers were attached to the same four locations used during the MRI/CT scanning. Then, the customized FUS helmet having an optical tracker was tightly secured on the subject’s head, and the virtual space of the neuroimage data was co-registered to the physical space of the subject’s head, as described in our previous work [16]. After the co-registration process, the location and orientation of the FUS transducers were adjusted for targeting the SI, as guided by the neuroimage data and the sonication planning (**Fig 3C and 3D**). Prior to establishing the connection to the BCI segment, FUS sonication (< 50 times) was administered to each side of the SI, and the subjects were instructed to report the type and location of the elicited tactile sensation using their own words. If necessary, the position of the FUS transducers were slightly maneuvered until the subject informed the operator about the presence of tactile sensations reliably from the contralateral hand/arm area to the side of sonication. The subjects were also instructed to respond only to the tactile sensations elicited from the hand in the subsequent BBI sessions.

Upon establishing the desired elicitation of the tactile sensation from the FUS stimulation, the BCI experimental staffs were notified and BBI experiment was jointly performed. BBI sessions were conducted three times, while CBI participant recorded the side (as left or right) of the elicited tactile sensations using touch sensors (pulse transducer MTL1010/D; ADInstruments, CO) attached on the index fingers. The timing of the triggering signal of the CBI was also simultaneously recorded using data acquisition hardware (PowerLab 4/35; ADInstruments, CO) and software (LabChart 7; ADInstruments, CO). After the BBI experiments, the participants of CBI procedure stayed in the hospital premises for additional 1 h to allow for assessment of post-sonication mental/physical status. No abnormal findings were revealed across all subjects during the assessment. Two months post-sonication telephone interview was also conducted as a follow-up for any discomforts or changes (associated with the sonication procedure) in health status experienced by the participants. None of them reported having any issues.

## Results

### Task performance from the BCI segment

The BCI portion of the BBI showed overall accuracy of 74.4 ± 15.9% (mean ± s.d., from *n* = 18 BCI sessions across the six participants) based on the left/right target-hit ratios (**Table 1**). The average accuracy of the BCI segment during three BBI sessions ranged from 93.3 ± 7.6% (BC6) to 50 ± 18% (BC2) among subjects. BC4 and BC6 showed 100% accuracy in some of the sessions, while the subject BC2 showed poor accuracy level of as low as 30%. Across the subjects and sessions, the target-miss occurred at a rate of 21.1 ± 18.0% (in a range of 0–14 trials out of 20 tasks; averaged from *n* = 18 grouped sessions across the six participants), while the aborted cases were on the order of 4.4 ± 5.7% (in a range of 0–4 trials out of 20 tasks). The average decision time for the BCI task was 1.13 ± 0.36 s.

**Table 1:** Accuracy and processing time of the BCI segment, CBI segment, and overall BBI communication for three sessions.

BCI segment				CBI segment				BBI communication				BBI link	
ID	Accuracy		Decision time (s)	ID	Accuracy		Response time (s)	ID	Accuracy		Delay (s)	Accuracy	
	Ratio	%			Ratio	%			Ratio	%		Ratio	%
**BC1**	16/20	80.0	1.219	**CB1**	19/19	100	0.455	**BB1**	16/20	80.0	0.232	16/16	100
	12/20	60.0	1.369		16/16	100	0.525		12/20	60.0	0.230	12/12	100
	16/20	80.0	1.581		18/18	100	0.529		16/20	80.0	0.230	16/16	100
	Mean	73.3	1.390		Mean	100	0.503		Mean	73.3	0.231	Mean	100
	s.d.	11.5	0.182		s.d.	0	0.041		s.d.	11.5	0.006	s.d.	0
**BC2**	6/20	30.0	0.519	**CB2**	19/19	100	0.288	**BB2**	5/20	25.0	0.018	5/6	83.3
	13/20	65.0	0.813		18/18	100	0.306		12/20	60.0	0.016	12/13	92.3
	11/20	55.0	0.575		20/20	100	0.342		11/20	55.0	0.021	11/11	100
	Mean	50.0	0.635		Mean	100	0.312		Mean	46.7	0.018	Mean	91.9
	s.d.	18.0	0.156		s.d.	0	0.027		s.d.	18.9	0.007	s.d.	8.3
**BC3**	18/20	90.0	0.881	**CB3**	19/19	100	0.581	**BB3**	18/20	90.0	0.045	18/18	100
	18/20	90.0	0.819		18/20	90.0	0.599		16/20	80.0	0.044	16/18	88.9
	12/20	60.0	1.363		17/18	94.4	0.580		13/20	65.0	0.041	12/12	100
	Mean	80.0	1.021		Mean	94.8	0.587		Mean	78.3	0.043	Mean	96.3
	s.d.	17.3	0.298		s.d.	5.0	0.011		s.d.	12.6	0.007	s.d.	6.4
**BC4**	20/20	100	1.056	**CB4**	17/20	85.0	0.867	**BB4**	17/20	85.0	0.058	17/20	85.0
	16/20	80.0	1.156		18/18	100	0.771		16/20	80.0	0.056	16/16	100
	16/20	80.0	1.038		18/19	94.7	0.758		16/20	80.0	0.056	16/16	100
	Mean	86.7	1.083		Mean	93.2	0.799		Mean	81.7	0.056	Mean	95.0
	s.d.	11.5	0.064		s.d.	7.6	0.060		s.d.	2.9	0.008	s.d.	8.7
**BC5**	14/20	70.0	1.394	**CB5**	20/20	100	0.619	**BB5**	14/20	70.0	0.204	14/14	100
	12/20	60.0	1.781		18/18	100	0.750		12/20	60.0	0.204	12/12	100
	12/20	60.0	1.719		19/19	100	0.679		12/20	60.0	0.208	12/12	100
	Mean	63.3	1.631		Mean	100	0.683		Mean	63.3	0.205	Mean	100
	s.d.	5.8	0.208		s.d.	0	0.066		s.d.	5.8	0.007	s.d.	0
**BC6**	17/20	85.0	1.125	**CB6**	18/18	100	0.987	**BB6**	15/20	75.0	0.221	15/17	88.2
	20/20	100	1.081		20/20	100	1.159		20/20	100	0.222	20/20	100
	19/20	95.0	0.906		20/20	100	1.097		19/20	95.0	0.221	19/19	100
	Mean	93.3	1.038		Mean	100	1.081		Mean	90.0	0.221	Mean	96.1
	s.d.	7.6	0.116		s.d.	0	0.087		s.d.	13.2	0.007	s.d.	6.8
	Grand	74.4	1.133		Grand	98.0	0.661		Grand	72.2	0.129	Grand	96.5
	s.d.	15.9	0.357		s.d.	3.1	0.252		s.d.	15.3	0.093	s.d.	3.1

The BCI accuracy was calculated as ‘(the number of hit trials) / (the total number of BCI task trials)’ within the BCI segment. CBI accuracy was calculated as ‘(the number of left/right directionally correct responses to the FUS) / (the total number of CBI trials)’ within the CBI segment. To examine the overall performance of the BBI communication, the accuracy was calculated as ‘(the number of successful BBI communication trials) / (the total number of BCI task trials)’. An additional index indicating the BBI link accuracy was calculated as ‘(the number of successful BBI communication trials) / (the number of hit trials in the BCI segment)’. Decision time, response time, and BBI delay were averaged from the total events in each segment/session.

### Task performance from the CBI segment

**Fig 4:**
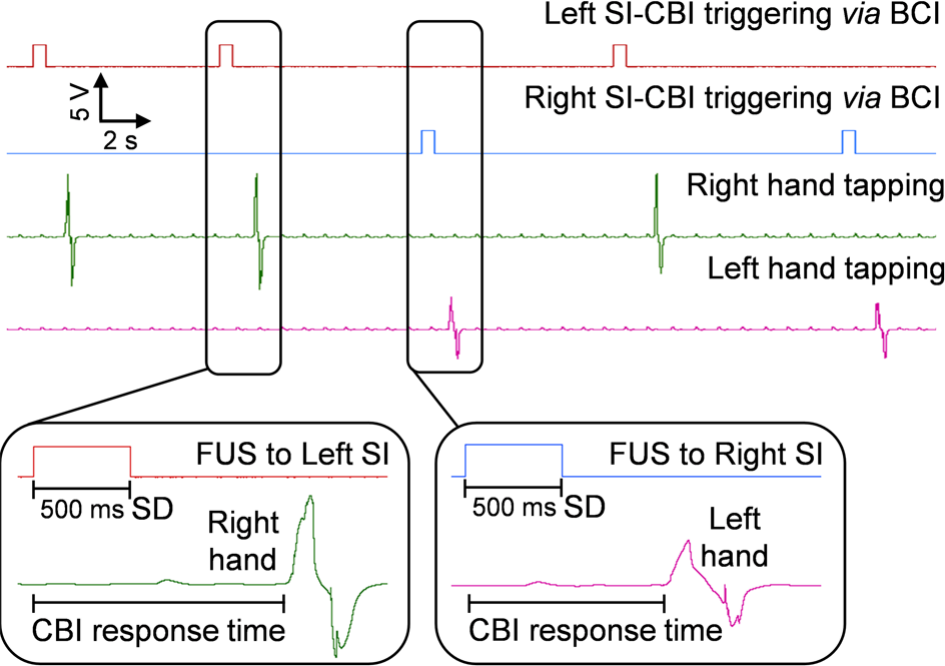
Exemplar view of CBI segment responses during a BBI session. FUS-mediated CBI segment exemplar capture screen of the BCI-originated trigger signals, which includes the elicited responses from the CBI segments.

An example of responses measured from a CBI segment is shown in **Fig 4**. The overall accuracy of FUS-mediated CBI segment was 98.0 ± 3.1% (mean ± s.d.) (**Table 1**). Among a total of six CBI subjects, four subjects (CB1, CB2, CB5, and CB6) achieved an accuracy of 100% across all three CBI sessions. Also, we observed few incidents (1–3 times) of non-responsiveness (CB3 and CB4) and false-responses (CB3) to the FUS sonication, rendering the overall accuracy less than perfect. This observation was in agreement with our previous experience, which is likely due to the slight changes of the sonication path during an experimental session [16]. Based on the measurement of subjects’ tapping response from the onset of FUS triggering signal (**Table 1**), the CBI response time to the FUS stimulation was 0.66 ± 0.25 s (*n* = 18 sessions).

### Paired performance of the BCI and CBI

Video recordings of an exemplar BBI procedure is shown in **S1 Video** (see **‘Supporting Information‘** section). Each pair’s accuracy during the BBI experiments was calculated as ‘the number of successful BBI communication trials’ divided by ‘the total number of BCI task trials (*i*.*e*., 20)’. Overall, the six pairs of subjects (named as ‘BB1’ through ‘BB6’ herein) showed the mean BBI accuracy of 72.2 ± 15.3% (mean ± s.d., six subject pairs across three sessions; **Table 1**). Additionally, to examine the ratio of correct transmission between BCI and CBI segments, another index (named ‘BBI link’ in **Table 1**) was defined as ‘the number of successful BBI communication trials’ divided by ‘the number of hit trials from the BCI segment’. The six pairs of subjects (‘BB1’ through ‘BB6’) showed the mean BBI link accuracy of 96.5 ± 3.1% (mean ± s.d., six subject pairs across three sessions; **Table 1**). Each pair achieved the link accuracy of 100% at least one session out of three. BB1 and BB5 showed the perfect link accuracy throughout all three BBI sessions while BB2 had the lowest mean link accuracy of 91.9 ± 8.3% (across 3 sessions). These high BBI link accuracies indicate that the results from the CBI segment did not happen by chance. For example, the probability of correctly guessing 17 out of 20 binary targets (even considering the worst CBI performance from session #1 of CB4; see **Table 1**) would be *p* = (1/2)^17^ < 10^−5^—which is extremely low to occur by chance. Also, all the accuracies measured from the experimental segments (BCI, CBI, and BBI) were significantly higher than the theoretical baseline of 50% (*t*-test, one-tailed, *p* < 10^−5^, *p* < 10^−32^, *p* < 10^−5^, respectively, *n* = 18 sessions).

### BBI transmission delay and averaged command generation per min

The transmission delay over the internet was 129 ± 93 ms (mean ± s.d., in a range of 16–232 ms), and the actuation of the FUS transducer (*i*.*e*., the triggering/onset timing of sonication) occurred 5.2 ± 2.6 ms (mean ± s.d., six CBI subjects across three sessions, data not shown) after the CBI control computer received the information from the BCI segment. The CBI subject’s tapping responses occurred in 661 ± 252 ms (mean ± s.d., six CBI subject across three sessions) from the onset timing of FUS triggering signal (**Table 1**). Overall, the BBI transmission delay was ~795 ms on average across the sessions (six subject pairs across three sessions). For the BBI communication, approximately eight commands were generated during one minute (on average) as the duration between the first and the last command received at the CBI segment from the BCI segment was ~2 min 35 s (a total of 20 commands were transmitted).

### Receiver Operating Characteristic (ROC) analysis

The behavioral data from each BCI/CBI segment as well as the BBI communication were categorized as a binary classification problem of left or right target, and examined by using a signal detection theory of Receiver Operating Characteristic (ROC) analysis [46]. As shown in **Table 2**, the task results of ‘True Right’, ‘False Right’, ‘True Left’, and ‘False Left’ were defined as ‘true positive (TP)’, ‘false positive (FP)’, ‘true negative (TN)’, and ‘false negative (FN)’, respectively. The example of these classifications is provided as a confusion matrix (**Table 2A**). We noticed few occurrences of computer transmission error within the CBI segment (noted as ‘COM error’, **Table 2B**). Aborted trials in the BCI segment and non-responsive/COM error cases in the CBI segment were all counted as ‘False (opposite side to the target)’ responses.

**Table 2:** Performance of the BCI segment, CBI segments, and overall BBI sessions.

(A)						Target side												
Result	Right					True Right (**TP**)							False Right (**FP**)					
	Left					False Left (**FN**)							True Left (**TN**)					
(B)																		
**BCI segment**						**CBI segment**							**BBI communication**					
**ID**	**TP**	**FN**	**FP**	**TN**	**AUC**	**ID**	**TP**	**FN**	**FP**	**TN**	**COM error**	**AUC**	**ID**	**TP**	**FN**	**FP**	**TN**	**AUC**
**BC1**	9	1	3	7	0.80	**CB1**	12	0	0	7	0	1.00	**BB1**	9	1	3	7	0.80
	8	2	6	4	0.60		11	0	0	5	0	1.00		8	2	6	4	0.60
	10	0	4	6	0.80		12	0	0	6	0	1.00		10	0	4	6	0.80
**BC2**	5	5	9	1	0.30	**CB2**	13	1	0	6	1	0.96	**BB2**	4	6	9	1	0.25
	7	3	4	6	0.65		9	2	0	9	2	0.91		6	4	4	6	0.60
	8	2	7	3	0.55		15	0	0	5	0	1.00		8	2	7	3	0.55
**BC3**	10	0	2	8	0.90	**CB3**	11	0	0	8	0	1.00	**BB3**	10	0	2	8	0.90
	9	1	1	9	0.90		9	1	1	9	0	0.90		8	2	2	8	0.80
	8	2	6	4	0.60		11	1	0	6	0	0.96		8	2	5	5	0.65
**BC4**	10	0	0	10	1.00	**CB4**	9	1	2	8	0	0.85	**BB4**	9	1	2	8	0.85
	10	0	4	6	0.80		12	0	0	6	0	1.00		10	0	4	6	0.80
	9	1	3	7	0.80		12	0	0	7	0	1.00		9	1	3	7	0.80
**BC5**	6	4	2	8	0.70	**CB5**	8	0	0	12	0	1.00	**BB5**	6	4	2	8	0.70
	4	6	2	8	0.60		6	0	0	12	0	1.00		4	6	2	8	0.60
	6	4	4	6	0.60		10	0	0	9	0	1.00		6	4	4	6	0.60
**BC6**	9	1	2	8	0.85	**CB6**	9	2	0	9	2	0.91	**BB6**	7	3	2	8	0.75
	10	0	0	10	1.00		10	0	0	10	0	1.00		10	0	0	10	1.00
	9	1	0	10	0.95		9	0	0	11	0	1.00		9	1	0	10	0.95
			Mean		0.74					Mean		0.97				Mean		0.72
			s.d.		0.18					s.d.		0.05				s.d.		0.18

(**A**) A confusion matrix was provided based on the binary classification of ‘Right’ and ‘Left’ targets. Each task result versus target side of the task was classified as ‘True Right-true positive (TP)’, ‘False Left-false negative’ (FN)’, ‘False Right-false positive (FP)’, and ‘True Left -true negative (TN)’. (**B**) The number of TP, FN, FP, and TN trials was tabulated for each BCI/CBI segment and for the overall BBI sessions. Area under the curve (AUC) of the ROC (shown in **Fig 5)** was also calculated for each segment and session. Computer transmission error occurred few times within the CBI segment (noted as ‘COM error’).

In the BCI segments, the accuracies on generating correct task results toward the target on the right (= TP / [TP + FN]) or left (= TN / [TN + FP]) were significantly different (*t*-test, one-tailed, *p* < 0.05, *n* = 18 sessions; 0.82 versus 0.67 in average), which may implicate the accuracy bias toward using right hand motor imagery. In case of the CBI segments, theses accuracies were not significantly different (*t*-test, one-tailed, *p* > 0.1, *n* = 18). Subsequently, overall BBI communication showed a statistically higher accuracy on generating responses from the left hand compared to the right (*t*-test, one-tailed, *p* < 0.05, *n* = 18; 0.78 versus 0.66 in average).

Based on **Table 2**, ROC graphs were depicted in a two dimensional coordinates system having the ‘TP rate’ as the *Y* axis and the ‘FP rate’ as *X* axis [46]. TP rate and FP rate were calculated using the definition of TP / (TP + FN) and FP / (FP + TN), respectively [46]. The ROC curves of BCI segment, CBI segment and overall BBI session (columns) were shown for all the subject pairs (numbered 1–6) across three BBI sessions in **Fig 5**and the area under the curve (AUC) was tabulated in **Table 2B**. As visualized in the ROC curves, the performance of the EEG-based BCI varied among the subjects and sessions (AUC = 0.74 ± 0.18; mean ± s.d., *n* = 18 sessions, in a range of 0.3–1.0). On the other hand, the performance in the FUS-mediated CBI segment was close to being a perfect classifier (AUC = 0.97 ± 0.05, *n* = 18, in a range of 0.85–1.0) across all participants. As a result, the overall subject-pair BBI performance became heavily dependent on the outcome of the BCI performance (AUC = 0.72 ± 0.18, *n* = 18, in a range of 0.25–1.0). For example, BBI pairs 2 and 5 showed overall low performance (AUC = 0.47 ± 0.19 and 0.63 ± 0.06, *n* = 3 sessions within each pair, respectively) due to the high FP rates from the BCI segments. However, the AUC values were significantly higher than the theoretical baseline of 0.5 across all the experimental segments (*t*-test, one-tailed, *p* < 10^−5^, *p* < 10^−30^, *p* < 10^−5^, respectively for BCI, CBI, and BBI segment; *n* = 18).

**Fig 5:**
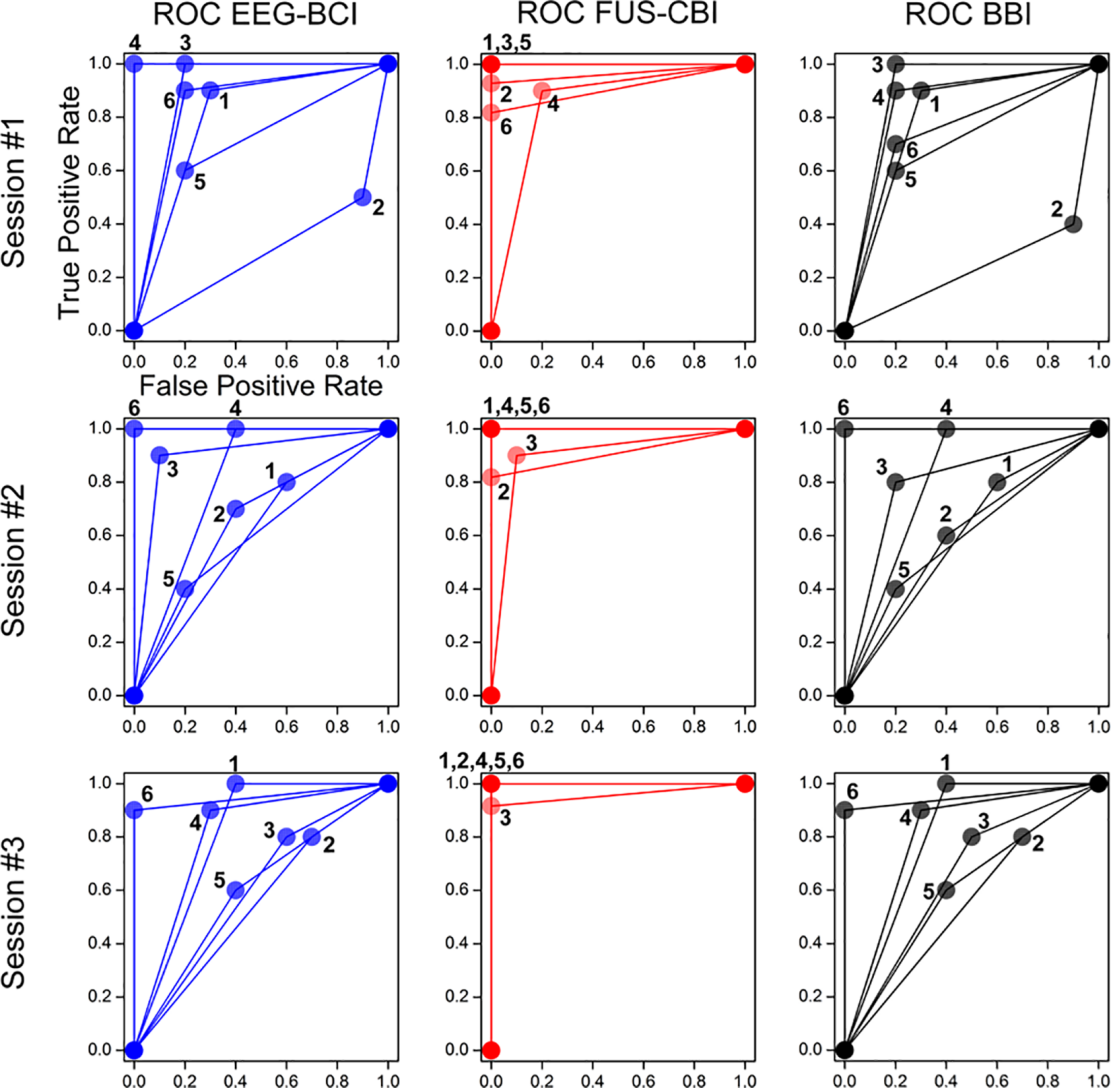
Performance of the six subject pairs for the implemented brain-to-brain interface (BBI). The ROC curves from the six subject pairs were shown in terms of EEG-based BCI task (left column), FUS-mediated CBI task (middle column), and overall BBI communication (right column). The rows indicate three multiple sessions (rows: session #1 to #3). Each BCI/CBI subject or BBI pair was labeled 1–6 around the data point circles.

## Discussion

TMS-based brain stimulation has been successfully deployed for non-invasive BBI in humans [14, 15, 24]. An alphabetical word has been encoded by a sender as binary codes using EEG, and TMS was used to subsequently stimulate the visual cortex of the receiver to induce phosphene perception, which can be decoded into a word [14]. TMS was also used to stimulate the receiver’s motor cortex (and subsequent induction of the finger motion) to transmit the sender’s intention to press a computer cursor [15]. More recent work demonstrated the collaborative interactions between two individuals established by hybrid use of non-invasive BBI (EEG and TMS) and conventional human computer machine interface (*via* the use of a computer mouse) in solving Twenty Question games [24].

We presented non-invasive cortical-level transmission of hemispheric brain function between two individuals that are remotely located. The sender’s choice of motor imagery of either left or right hand activated the corresponding motor areas in each hemisphere and the associated changes in EEG *mu* rhythm were detected. Consequently, the intention was transmitted over the internet to stimulate the receiver’s somatosensory area, informing the intention of the sender. Although there were few incidents of missed transmission of the information (noted as ‘COM error’ in **Table 2**), the FUS-driven CBI was able to stimulate the hemispheric SI, resulting in a high rate of successful BBI transmissions (*i*.*e*., the BBI link accuracy of 91.9 ± 8.3%). The sonication procedure did not show any adverse effects such as discomforts or mental changes across the subjects.

We believe that this is the first demonstration of using FUS-based brain stimulation among humans in the context of BBI. Small FUS transducers effectively allowed for the independent administration of stimulatory ultrasonic waves to the SI of each hemisphere, linking the hemispheric brain activities between the two individuals. A similar implementation (*i*.*e*., stimulating two separate hemispheric brain areas) using multiple TMS coils (and subsequent BBI application) would have been more difficult to achieve because the strong electromagnetic field generated by the TMS may interfere/distort the magnetic field of one another, not to mention the potential mutual interactions between the magnetic force generated by the coils.

Despite the selection of the participants who showed relatively high task accuracies (≥ 70%) during the BCI test sessions, only seven out of 29 subjects were able to meet the criteria. Although different methods and task difficulties are known to affect the accuracy of the BCI task, the ratio of competent participants from this study was smaller than most of the previously reported value (~20% incompetence rate) [47]. This so-called ‘BCI illiteracy’ problem, the presence of individuals who tend to show low BCI performance accuracy, is a known challenge in BCI research [47]. In the BCI segment, the participants showed superior classification accuracy when engaging in motor-imagery of their dominant hand (*i*.*e*., all the BCI subjects were right-handed), which may implicate the role of handedness in performing the BCI tasks. However, sufficient adaptation of the task schemes (by the subjects), longer training sessions and investment on time and details of parameter optimization, combined with the use of novel machine-learning techniques [47], may help to improve the accuracy of the BCI task.

Among the participants in the BCI segment during the BBI implementation, individual- and session-specific variability existed in terms of the task accuracy (example shown in the **Table 1**). The EEG-mediated BCI segment showed overall accuracy on the order of 75% (**Table 1**), ranging 63.3–93.3% in most of the participants except one (*i*.*e*., the subject ‘BC2’), who missed many targets in the first session (thus showing 30% accuracy). To the contrary, the CBI segment showed far greater accuracy compared to the BCI segment. Most of the subjects were able to show greater than 93% accuracy (most of them reaching 100% accuracy). We believe that maneuvering the transducer location to confirm the elicitation of tactile sensation (prior to the actual BBI session) contributed to this high response accuracy [16]. Few skips in internet-mediated transmission was also noted from two subject pairs (BBI2 and BBI6, **Table 2**), but we believe it was a temporary hardware/software related error that could have been prevented by testing/assuring the establishment of adequate communication prior to the experiment.

Based on the inherent delays in subject’s tapping action of a finger after the perception of the tactile sensations of the hand (measured to be ~200 ms after the sensing of the elicited responses or to the any external stimulus) [16], the true latency in FUS-mediated CBI is anticipated to be much shorter. Overall, for the BBI transmission, about eight commands were generated and transmitted during one minute period in average, suggesting the bandwidth of the transmission. Potential maximum transmission rate, as defined by the limitation of the BCI task (inter-trial-intervals of 7 s; **Fig 2**) was approximately 10 commands per minute, however, the overall transmission rate was slightly reduced by missed or false responses. Increasing the sensitivity of the BCI detection method would potentially increase the transmission rate.

Unlike the TMS devices that may interfere with EEG data acquisition, the use of acoustic brain stimulation modality would enable the concomitant use of EEG for BCI applications. For example, FUS has been successfully deployed simultaneously with EEG and MRI environment to characterize the brain responses from the FUS stimulation in animals and humans [17, 43]. This provides a fascinating possibility to implement both EEG-BCI and FUS-CBI to a single individual. We believe this capability may confer bidirectional exchange of neural signals among individuals, enabling personal interactions to occur through thought processes only. For example, this may eliminate the need for having computer-mouse mediated responses from the previous Twenty Question BBI experiment [24]. On a similar note, simultaneous use of BCI and CBI within a single individual would also open up the possibility of closed-loop feedback of one’s own brain activity. One of the immediate clinical utilities of such an approach would be the on-line detection of the focal epileptic seizure (*via* surface or implanted electrodes) and its on-demand, localized suppression using FUS. FUS-mediated brain stimulation also casts interesting possibilities in modulating deep brain structures for the brain-to-brain communication where the utility of deep brain stimulation (DBS) has been suggested [48].

The individual MRI/fMRI data was used to guide the sonication target in the present study. The finger tapping tasks used in the fMRI was effective in guiding sonication to the SI area; however, adoption of the tactile-only fMRI stimulation paradigm, for example, as reported by Schweisfurth and colleagues [49], will be helpful to localize the SI areas with potential mapping of their functional sub-regions. As the size of the focus is small while the individual functional neuroanatomy varies greatly, we believe that the image-guidance system was absolutely needed to limit the unnecessary exposure of the brain to the FUS. In future studies, however, it is also conceivable that heuristic placement of the transducer and adjustment of acoustic intensity may be possible until the desired neural response is observed. This approach should only be conducted with caution after establishing the safety profile regarding the repeated sonication (*i*.*e*., similar to the case of TMS whereby the location/orientation of the TMS coil and applied magnetic intensity is adjusted for each individual).

We note that FUS was directed to the SI and elicited tactile sensations that were perceived by the participants. This is distinguished from eliciting direct motor responses that were employed by Rao and colleagues in their TMS-mediated BBI implementation [15, 21, 24]. The current method relies on inducing peripheral sensation of the innervated hand SI areas of the brain. Most of the subjects did not receive any additional tactile sensation resulting from the sonication, for example, the scalp tactile sensation that is quite common in TMS. This finding is congruent with our previous investigation of the SI and V1 stimulation [16, 17]. However, few subjects (two) did report hearing a ‘beeping’ sound on the opposite hemispheric side of the sonication. We conjecture that the sonication that passed through the SI reached the inner ear of the opposite hemisphere, inducing the perception of the beeping sound, as there has been a report of FUS being used to stimulate the inner ear structure [50]. It is also plausible that the higher acoustic intensity used in this experiment (*i*.*e*., 35.0 W/cm^2^ I_sppa_ versus 3.0 W/cm^2^ I_sppa_ in the past) could have attributed to this auditory perception. Previously, Tyler and colleagues [18], which used a relatively high incident acoustic intensity (23.87 W/cm^2^ I_sppa_) reported the occurrence of a similar ‘chirping’ noise that was reported by the subject, but indistinguishable to those experienced during the sham FUS condition. These occurred in the absence of the tactile sensations at the scalp.

We do not believe, however, that the presence of this extra peripheral perception (*e*.*g*., beeping sounds) could have affected the results of the study since (1) the sounds were perceived only by few individuals, and (2) all individuals were instructed to respond only to the tactile sensations elicited from the hand. Nonetheless, the conscious perception of these sensations could have introduced a degree of confounders during the establishment of direct neural interfaces. Therefore, direct stimulation of the motor cortex (M1) and elicitation of unconscious motor response among the CBI participants would be needed for enabling ‘unconscious’ and ‘direct’ BBI. FUS-mediated stimulation of the motor area and elicitation of overt muscle recruitment/motion have not been demonstrated among humans. Further investigations are urgently needed to establish proper safety guidelines as well as effective sonication parameters to elicit direct motor responses.

In the present study, we used either left of right motor imagery to generate the BBI commands. It represents a rudimentary 2-bit encoding scheme, depending on the stimulation of either or both hemispheres. Based on our recent study in which FUS was used to stimulate visual cortex while inducing phosphene perception [17], expansion of the additional dimension of the encoding system is feasible if one could add visual stimulation as an independent encoding option for the CBI (*e*.*g*., 3-bit encoding). Presuming that lateralized, independent stimulation of the each visual hemisphere (*i*.*e*., left or right) is possible, it will add another degree of freedom in encoding (*i*.*e*., 4-bit encoding), thus, further increasing the potential bandwidth of communication. However, the mutual interference (such as beam crossing within the cranial cavity) between ultrasound waves is possible, calling for caution against unwanted formation of unusually high levels of acoustic intensity. Further study is needed to examine the possibility and safety of such approaches.

Increasing the information detected by the EEG (perhaps through high-resolution EEG source mapping) [51, 52], combined with use of selective/simultaneous operation of multiple FUS transducers, would expand the overall bandwidth of the transmission. The bidirectional connectivity, augmented with the increase in bandwidth in neural communication, casts interesting possibility—one example was recently demonstrated by Nicolelis and colleagues whereby the cell-level recordings and stimulation effectively inter-connected the neural functions of rodents in jointly performing visual or motor tasks [53]. The expansion of the information bandwidth and mutual exchange of the neural information, all in non-invasive fashion, constitutes subjects of future investigation. The overall impact of such BBI on human behavior remains to be elucidated.

## Supporting information

S1 Video The video recordings of BBI procedure. The EEG data for the BCI procedure is not currently available for public access due to privacy concerns, as enforced by the Institutional Review Board of Korea Institute of Science and Technology (KIST). However, individual, case-by-case access to the data is available *via* request, and interested parties may request the data to Research Support Department of KIST (shcho@kist.re.kr) with the approval number (2014–008). All other relevant data are within the paper and its Supporting Information file.
(AVI)

## References

[1] Alexander GM, Rogan SC, Abbas AI, Armbruster BN, Pei Y, Allen JA, et al Remote control of neuronal activity in transgenic mice expressing evolved G protein-coupled receptors. Neuron. 2009;63(1):27–39. doi: 10.1016/j.neuron.2009.06.014 19607790 10.1016/j.neuron.2009.06.014 PMC2751885

[2] Fanselow EE, Nicolelis MA. Behavioral modulation of tactile responses in the rat somatosensory system. J Neurosci. 1999;19(17):7603–16. 10460266 10.1523/JNEUROSCI.19-17-07603.1999 PMC6782523

[3] Laubach M, Wessberg J, Nicolelis MA. Cortical ensemble activity increasingly predicts behaviour outcomes during learning of a motor task. Nature. 2000;405(6786):567–71. doi: 10.1038/35014604 10850715 10.1038/35014604

[4] Nicolelis MA, Baccala LA, Lin RC, Chapin JK. Sensorimotor encoding by synchronous neural ensemble activity at multiple levels of the somatosensory system. Science. 1995;268(5215):1353–8. 7761855 10.1126/science.7761855

[5] Pais-Vieira M, Lebedev M, Kunicki C, Wang J, Nicolelis MAL. A brain-to-brain interface for real-time sharing of sensorimotor information. Sci Rep. 2013;3:1319 doi: 10.1038/srep01319 23448946 10.1038/srep01319 PMC3584574

[6] Carmena JM, Lebedev MA, Crist RE, O'Doherty JE, Santucci DM, Dimitrov DF, et al Learning to control a brain-machine interface for reaching and grasping by primates. PLoS Biol. 2003;1(2):E42 doi: 10.1371/journal.pbio.0000042 14624244 10.1371/journal.pbio.0000042 PMC261882

[7] Chapin JK, Moxon KA, Markowitz RS, Nicolelis MA. Real-time control of a robot arm using simultaneously recorded neurons in the motor cortex. Nat Neurosci. 1999;2(7):664–70. doi: 10.1038/10223 10404201 10.1038/10223

[8] Nicolelis MAL, Dimitrov D, Carmena JM, Crist R, Lehew G, Kralik JD, et al Chronic, multisite, multielectrode recordings in macaque monkeys. Proc Natl Acad Sci U S A. 2003;100(19):11041–6. doi: 10.1073/pnas.1934665100 12960378 10.1073/pnas.1934665100 PMC196923

[9] O’Doherty JE, Lebedev MA, Ifft PJ, Zhuang KZ, Shokur S, Bleuler H, et al Active tactile exploration using a brain-machine-brain interface. Nature. 2011;479(7372):228–31. doi: 10.1038/nature10489 21976021 10.1038/nature10489 PMC3236080

[10] Wessberg J, Stambaugh CR, Kralik JD, Beck PD, Laubach M, Chapin JK, et al Real-time prediction of hand trajectory by ensembles of cortical neurons in primates. Nature. 2000;408(6810):361–5. doi: 10.1038/35042582 11099043 10.1038/35042582

[11] Fabiani GE, McFarland DJ, Wolpaw JR, Pfurtscheller G. Conversion of EEG activity into cursor movement by a brain-computer interface (BCI). IEEE Trans Neural Syst Rehabil Eng. 2004;12(3):331–8. doi: 10.1109/TNSRE.2004.834627 15473195 10.1109/TNSRE.2004.834627

[12] Mellinger J, Schalk G, Braun C, Preissl H, Rosenstiel W, Birbaumer N, et al An MEG-based brain-computer interface (BCI). Neuroimage. 2007;36(3):581–93. doi: 10.1016/j.neuroimage.2007.03.019 17475511 10.1016/j.neuroimage.2007.03.019 PMC2017111

[13] Yoo S-S, Fairneny T, Chen N-K, Choo S-E, Panych LP, Park H, et al Brain-computer interface using fMRI: spatial navigation by thoughts. Neuroreport. 2004;15(10):1591–5. 15232289 10.1097/01.wnr.0000133296.39160.fe

[14] Grau C, Ginhoux R, Riera A, Nguyen TL, Chauvat H, Berg M, et al Conscious brain-to-brain communication in humans using non-invasive technologies. PLoS One. 2014;9(8):e105225 doi: 10.1371/journal.pone.0105225 25137064 10.1371/journal.pone.0105225 PMC4138179

[15] Rao RPN, Stocco A, Bryan M, Sarma D, Youngquist TM, Wu J, et al A direct brain-to-brain interface in humans. PLoS One. 2014;9(11):e111332 doi: 10.1371/journal.pone.0111332 25372285 10.1371/journal.pone.0111332 PMC4221017

[16] Lee W, Kim H, Jung Y, Song I-U, Chung YA, Yoo S-S. Image-guided transcranial focused ultrasound stimulates human primary somatosensory cortex. Sci Rep. 2015;5:8743 doi: 10.1038/srep08743 25735418 10.1038/srep08743 PMC4348665

[17] Lee W, Kim H-C, Jung Y, Chung YA, Song I-U, Lee J-H, et al Transcranial focused ultrasound stimulation of human primary visual cortex. Sci Rep. 2016;6:34026 doi: 10.1038/srep34026 27658372 10.1038/srep34026 PMC5034307

[18] Legon W, Sato TF, Opitz A, Mueller J, Barbour A, Williams A, et al Transcranial focused ultrasound modulates the activity of primary somatosensory cortex in humans. Nat Neurosci. 2014;17(2):322–9. doi: 10.1038/nn.3620 24413698 10.1038/nn.3620

[19] Min B-K, Marzelli MJ, Yoo S-S. Neuroimaging-based approaches in the brain–computer interface. Trends Biotechnol. 2010;28(11):552–60. doi: 10.1016/j.tibtech.2010.08.002 20810180 10.1016/j.tibtech.2010.08.002

[20] Nicolelis M. Beyond boundaries: the new neuroscience of connecting brains with machines—and how it will change our lives: Macmillan; 2011.

[21] Rao RPN, Stocco A. When two brains connect. Sci Am Mind. 2014;25(6):36–9.

[22] Yoo S-S, Kim H, Filandrianos E, Taghados SJ, Park S. Non-invasive brain-to-brain interface (BBI): establishing functional links between two brains. PLoS One. 2013;8(4):e60410 doi: 10.1371/journal.pone.0060410 23573251 10.1371/journal.pone.0060410 PMC3616031

[23] Li G, Zhang D. Brain-computer interface controlled cyborg: establishing a functional information transfer pathway from human brain to cockroach brain. PLoS One. 2016;11(3):e0150667 doi: 10.1371/journal.pone.0150667 26982717 10.1371/journal.pone.0150667 PMC4794219

[24] Stocco A, Prat CS, Losey DM, Cronin JA, Wu J, Abernethy JA, et al Playing 20 questions with the mind: collaborative problem solving by humans using a brain-to-brain interface. PLoS One. 2015;10(9):e0137303 doi: 10.1371/journal.pone.0137303 26398267 10.1371/journal.pone.0137303 PMC4580467

[25] Sliwinska MW, Vitello S, Devlin JT. Transcranial magnetic stimulation for investigating causal brain-behavioral relationships and their time course. J Vis Exp. 2014;7 2014(89):e51735. 10.3791/51735 PMC4219631 25079670

[26] Bien N, ten Oever S, Goebel R, Sack AT. The sound of size: crossmodal binding in pitch-size synesthesia: a combined TMS, EEG and psychophysics study. Neuroimage. 2012;59(1):663–72. doi: 10.1016/j.neuroimage.2011.06.095 21787871 10.1016/j.neuroimage.2011.06.095

[27] Peterchev AV, Wagner TA, Miranda PC, Nitsche MA, Paulus W, Lisanby SH, et al Fundamentals of transcranial electric and magnetic stimulation dose: Definition, selection, and reporting practices. Brain Stimul. 2012;5(4):435–53. doi: 10.1016/j.brs.2011.10.001 22305345 10.1016/j.brs.2011.10.001 PMC3346863

[28] Ruff CC, Blankenburg F, Bjoertomt O, Bestmann S, Freeman E, Haynes J-D, et al Concurrent TMS-fMRI and psychophysics reveal frontal influences on human retinotopic visual cortex. Curr Biol. 2006;16(15):1479–88. doi: 10.1016/j.cub.2006.06.057 16890523 10.1016/j.cub.2006.06.057

[29] Groppa S, Werner-Petroll N, Münchau A, Deuschl G, Ruschworth MFS, Siebner HR. A novel dual-site transcranial magnetic stimulation paradigm to probe fast facilitatory inputs from ipsilateral dorsal premotor cortex to primary motor cortex. Neuroimage. 2012;62(1):500–9. doi: 10.1016/j.neuroimage.2012.05.023 22626848 10.1016/j.neuroimage.2012.05.023

[30] Hartwigsen G, Price CJ, Baumgaertner A, Geiss G, Koehnke M, Ulmer S, et al The right posterior inferior frontal gyrus contributes to phonological word decisions in the healthy brain: Evidence from dual-site TMS. Neuropsychologia. 2010;48(10):3155–63. doi: 10.1016/j.neuropsychologia.2010.06.032 20600177 10.1016/j.neuropsychologia.2010.06.032 PMC3223523

[31] Krasovitski B, Frenkel V, Shoham S, Kimmel E. Intramembrane cavitation as a unifying mechanism for ultrasound-induced bioeffects. Proc Natl Acad Sci U S A. 2011;108(8):3258–63. doi: 10.1073/pnas.1015771108 21300891 10.1073/pnas.1015771108 PMC3044354

[32] Plaksin M, Kimmel E, Shoham S. Cell-type-selective effects of intramembrane cavitation as a unifying theoretical framework for ultrasonic neuromodulation. eNeuro. 2016;22(3):ENEURO.0136-15.2016. 10.1523/ENEURO.0136-15.2016 PMC4917736 27390775

[33] Plaksin M, Shoham S, Kimmel E. Intramembrane cavitation as a predictive bio-piezoelectric mechanism for ultrasonic brain stimulation. Phys Rev X. 2014;4(1):011004.

[34] Deffieux T, Younan Y, Wattiez N, Tanter M, Pouget P, Aubry J-F. Low-intensity focused ultrasound modulates monkey visuomotor behavior. Curr Biol. 2013;23(23):2430–3. doi: 10.1016/j.cub.2013.10.029 24239121 10.1016/j.cub.2013.10.029

[35] Lee W, Chung YA, Jung Y, Song IU, Yoo SS. Simultaneous acoustic stimulation of human primary and secondary somatosensory cortices using transcranial focused ultrasound. BMC Neurosci. 2016;17(1):68 doi: 10.1186/s12868-016-0303-6 27784293 10.1186/s12868-016-0303-6 PMC5081675

[36] Oldfield RC. The assessment and analysis of handedness: The Edinburgh inventory. Neuropsychologia. 1971;9(1):97–113. 5146491 10.1016/0028-3932(71)90067-4

[37] Pfurtscheller G, Brunner C, Schlögl A, Lopes da Silva FH. Mu rhythm (de)synchronization and EEG single-trial classification of different motor imagery tasks. Neuroimage. 2006;31(1):153–9. doi: 10.1016/j.neuroimage.2005.12.003 16443377 10.1016/j.neuroimage.2005.12.003

[38] Schalk G, McFarland DJ, Hinterberger T, Birbaumer N, Wolpaw JR. BCI2000: a general-purpose brain-computer interface (BCI) system. IEEE Trans Biomed Eng. 2004;51(6):1034–43. doi: 10.1109/TBME.2004.827072 15188875 10.1109/TBME.2004.827072

[39] Wolpaw JR, Birbaumer N, McFarland DJ, Pfurtscheller G, Vaughan TM. Brain-computer interfaces for communication and control. Clin Neurophysiol. 2002;113(6):767–91. 12048038 10.1016/s1388-2457(02)00057-3

[40] Teplan M. Fundamentals of EEG measurement. Meas Sci Rev. 2002;2(2):1–11.

[41] Maes F, Collignon A, Vandermeulen D, Marchal G, Suetens P. Multimodality image registration by maximization of mutual information. IEEE Trans Med Imaging. 1997;16(2):187–98. doi: 10.1109/42.563664 9101328 10.1109/42.563664

[42] Lee W, Lee SD, Park MY, Yang J, Yoo S-S. Evaluation of polyvinyl alcohol cryogel as an acoustic coupling medium for low-intensity transcranial focused ultrasound. Int J Imaging Syst Technol. 2014;24(4):332–8.

[43] Yoo S-S, Bystritsky A, Lee J-H, Zhang Y, Fischer K, Min B-K, et al Focused ultrasound modulates region-specific brain activity. Neuroimage. 2011;56(3):1267–75. doi: 10.1016/j.neuroimage.2011.02.058 21354315 10.1016/j.neuroimage.2011.02.058 PMC3342684

[44] McDannold N, Vykhodtseva N, Jolesz FA, Hynynen K. MRI investigation of the threshold for thermally induced blood–brain barrier disruption and brain tissue damage in the rabbit brain. Magn Reson Med. 2004;51(5):913–23. doi: 10.1002/mrm.20060 15122673 10.1002/mrm.20060

[45] Teschan P, Gellhorn E. Influence of increased temperature on activity of the cerebral cortex. Am J Physiol. 1949;159(1):1–5. 15391069 10.1152/ajplegacy.1949.159.1.1

[46] Fawcett T. An introduction to ROC analysis. Pattern Recognit Lett. 2006;27(8):861–74.

[47] Vidaurre C, Blankertz B. Towards a cure for BCI illiteracy. Brain Topogr. 2010;23(2):194–8. doi: 10.1007/s10548-009-0121-6 19946737 10.1007/s10548-009-0121-6 PMC2874052

[48] Glannon W, Ineichen C. Chapter 19—Philosophical Aspects of Closed-Loop Neuroscience In: Hady AE, editor. Closed Loop Neuroscience. San Diego: Academic Press; 2016 p. 259–70.

[49] Schweisfurth MA, Frahm J, Schweizer R. Individual fMRI maps of all phalanges and digit bases of all fingers in human primary somatosensory cortex. Front Hum Neurosci. 2014;8:658 doi: 10.3389/fnhum.2014.00658 25228867 10.3389/fnhum.2014.00658 PMC4151507

[50] Gavrilov LR. Use of focused ultrasound for stimulation of nerve structures. Ultrasonics. 1984;22(3):132–8. 6372189 10.1016/0041-624x(84)90008-8

[51] Ermer JJ, Mosher JC, Baillet S, Leah RM. Rapidly recomputable EEG forward models for realistic head shapes. Phys Med Biol. 2001;46(4):1265–81. 11324964 10.1088/0031-9155/46/4/324

[52] Hassan M, Dufor O, Merlet I, Berrou C, Wendling F. EEG source connectivity analysis: from dense array recordings to brain networks. PLoS One. 2014;9(8):e105041 doi: 10.1371/journal.pone.0105041 25115932 10.1371/journal.pone.0105041 PMC4130623

[53] Pais-Vieira M, Chiuffa G, Lebedev M, Yadav A, Nicolelis MAL. Building an organic computing device with multiple interconnected brains. Sci Rep. 2015;5:11869 doi: 10.1038/srep11869 26158615 10.1038/srep11869 PMC4497302

